# Epigenome-Wide Association Study in Asian Cohort Identifies Novel DNA Methylation Markers for Carotid Intima-Media Thickness

**DOI:** 10.21203/rs.3.rs-9046066/v1

**Published:** 2026-03-16

**Authors:** Konstanze Tan, Sarah E Harris, Jane Maddock, Darwin Tay, Pritesh R Jain, The HELIOS Study Team, Shi Qi Mok, Maxime Herbrard, Ulf Schminke, Can Can Xue, Liuh Ling Goh, Theresia H Mina, Alexander Teumer, Weng Khong Lim, Khai Pang Leong, Khung Keong Yeo, Ching-Yu Cheng, Xueling Sim, Lee Eng Sing, Joanna M Wardlaw, Henry Völzke, Andrew Wong, Simon R Cox, Rinkoo Dalan, Abbas Dehghan, Marie Loh

**Affiliations:** Lee Kong Chian School of Medicine, Nanyang Technological University Singapore, Singapore; Lothian Birth Cohorts, Department of Psychology, University of Edinburgh Edinburgh, United Kingdom; Division of Surgery and Interventional Science, University College London London, United Kingdom; Lee Kong Chian School of Medicine, Nanyang Technological University Singapore, Singapore; Lee Kong Chian School of Medicine, Nanyang Technological University Singapore, Singapore; Lee Kong Chian School of Medicine, Nanyang Technological University Singapore, Singapore; Laboratory of Complex Disease Genetics, Genome Institute of Singapore, Agency for Science, Technology and Research, Singapore, Singapore; NPM-Genomic Intelligence & Informatics Engine (NPM-GINIE), Genome Institute of Singapore, Agency for Science, Technology and Research, Singapore, Singapore; Department of Neurology, University Medicine Greifswald, Greifswald, Germany; Singapore Eye Research Institute, Singapore National Eye Centre, Singapore; Personalised Medicine Service, Tan Tock Seng Hospital, Singapore, Singapore; Lee Kong Chian School of Medicine, Nanyang Technological University Singapore, Singapore; Department of Psychiatry and Psychotherapy, University Medicine Greifswald, Greifswald, Germany; SingHealth Duke-NUS Institute of Precision Medicine, Singapore, 169609, Singapore; SingHealth Duke-NUS Genomic Medicine Centre, Singapore, 168582, Singapore; Cancer & Stem Cell Biology Program, Duke-NUS Medical School, Singapore, 169857, Singapore; Laboratory of Genome Variation Analytics, Genome Institute of Singapore, Agency for Science, Technology and Research, Singapore, 138672, Singapore; Personalised Medicine Service, Tan Tock Seng Hospital, Singapore, Singapore; SingHealth Duke-NUS Institute of Precision Medicine, Singapore, 169609, Singapore; Department of Cardiology, National Heart Centre Singapore, Singapore, 169609, Singapore; Duke-NUS Medical School, Singapore, 169857, Singapore; Singapore Eye Research Institute, Singapore National Eye Centre, Singapore; Center for Innovation and Precision Eye Health, Yong Loo Lin School of Medicine, National University of Singapore, Singapore; Department of Ophthalmology, Yong Loo Lin School of Medicine, National University of Singapore, Singapore; Saw Swee Hock School of Public Health, National University of Singapore and National University Health System, 117549, Singapore; Lee Kong Chian School of Medicine, Nanyang Technological University Singapore, Singapore; Centre for Clinical Brain Sciences, UK Dementia Research Institute, University of Edinburgh, Edinburgh, UK; Institute for Community Medicine, University Medicine Greifswald Greifswald, Germany; Unit for Lifelong Health and Ageing, MRC National Survey of Health and Development, University College London London, United Kingdom; Lothian Birth Cohorts, Department of Psychology, University of Edinburgh Edinburgh, United Kingdom; Senior Consultant, Department of Endocrinology, Tan Tock Seng Hospital, National Healthcare Group, Singapore, Singapore; Associate Professor & Co-Director Metabolism, Nutrition and Health, Lee Kong Chian School of Medicine, Nanyang Technological University, Singapore, Singapore; School of Public Health, Faculty of Medicine, Imperial College London, United Kingdom; MRC Centre for Environment and Health, Imperial College London, United Kingdom; Dementia Research Institute, Imperial College London London, United Kingdom; Lee Kong Chian School of Medicine, Nanyang Technological University Singapore, Singapore

**Keywords:** Carotid intima-media thickness (cIMT), DNA methylation, Epigenome-wide association study (EWAS), Asian, atherosclerosis

## Abstract

**Background:**

Carotid-intima media-thickness (cIMT) predicts cardiovascular events and informs mechanistic research on cardiovascular diseases. However, cardiovascular disease research remains Eurocentric despite etiological differences across ancestries. Incorporating Asian populations who face substantial cardiovascular disease burden with distinct etiological landscape can enhance our understanding of cIMT biology and subclinical processes linked to CVD. This study aims to elucidate methylation-based mechanisms of cIMT through DNA methylation profiling integrated with multi-omics data and clinically informative cIMT thresholds, leveraging an Asian cohort to enhance discovery.

**Methods:**

We conducted an epigenome-wide association study (EWAS) of cIMT using peripheral blood DNA methylation at ~850,000 CpG sites in the Asian Health for Life in Singapore (HELIOS) cohort (n=1,357), followed by targeted trans-ancestry meta-analysis with European cohorts (overall n=2,765). Causal inference analyses (summary data-based Mendelian Randomisation [SMR] and colocalisation) evaluated methylation-mediated effects on cIMT, cardiovascular disease and proximal gene expression. We derived a methylation risk score (MRS) and tested its association with cIMT thresholds indicative of elevated cardiovascular risk (≥75^th^ percentile for age, sex and ethnicity).

**Results:**

Three novel CpG-cIMT associations were identified (P<9.35E-07). Causal analyses supported cg08227773 methylation-mediated effects on both coronary artery disease risk (P_SMR_=2.91E-05, coloc PP.H4 =0.91) and *NBEAL2* (Neurobeachin-like 2) expression (P_SMR_=9.13E-08, coloc PP.H4=0.69), a gene implicated in immune dysregulation. MRS of cIMT aggregating the three sentinel CpGs was associated with clinically-informative cIMT elevation (Odds Ratio=2.75 for Q4 vs Q1, 95% CI: 1.47–5.13).

**Conclusions:**

Through Asian-led discovery, this study identifies three novel DNA methylation markers for cIMT that are linked to cIMT elevation above clinically meaningful risk thresholds. Causal inference analyses suggest methylation-mediated coronary artery disease risk via *NBEAL2* regulation, nominating biologically relevant targets while underscoring the need for larger multi-omics resources to refine mechanisms.

## 4. INTRODUCTION

Cardiovascular disease (CVD) is the leading global cause of death and disability-adjusted life years.(1) Preceding the overt manifestation of clinical CVD events in adulthood are lifelong atherogenic changes in youth.(2) Correspondingly, carotid intima media thickness (cIMT), a non-invasive ultrasound measure of atherosclerotic burden reflecting cumulative arterial remodeling, allows CVD to be studied in the subclinical phase. (3, 4, 5, 6) cIMT robustly predicts incident myocardial infarction and stroke, correlates with established CVD risk factors, and also shows genetic correlation with CVD outcomes.(7, 8, 9, 10, 11, 12) However, ancestry-related differences in effect sizes of risk-factor associations and modest SNP-heritability estimates (26%−28%) point to the contribution of additional regulatory mechanisms of cIMT.(9, 10)

DNA methylation, the covalent addition of a methyl group to the 5’ carbon of cytosine in CpG dinucleotides, integrates cumulative inherited and environmental exposures, representing a practical molecular tool to interrogate cIMT biology.(13) The first published epigenome-wide association study (EWAS) of cIMT, a European multi-cohort meta-analysis (8 cohorts; n=6,400; 450K array), identified an epigenome-wide significant signal at the canonical smoking biomarker, cg05575921 (*AHRR*) along with 33 other genomic regions.(14) A second published EWAS of cIMT in the Dominican Republic (61 families; 769 individuals; EPIC array) reported methylation alterations at the *PM20D1* promoter, implicating mitochondrial uncoupling and reactive oxygen species generation.(15) Crucially, cg05575921 did not replicate in the Dominican Republic study.

Motivated by the value of ancestral diversity for elucidating cIMT biology, we focus on Asian populations, whose etiologic architecture of cIMT adds complementary insight into arterial remodeling.(9, 16) We undertook a two-tier EWAS strategy: first, we meta-analysed ~850,000 CpG across Chinese, Malays and Indian participants from the Health for Life in Singapore (HELIOS) cohort (n=1,357) for an Asian-focused screen.(17) Signals demonstrating preliminary relevance were then advanced to trans-ancestry meta-analysis with European cohorts. Robustly associated CpG sites were subsequently analysed through integrative omics approaches, then combined into a methylation risk score (MRS) for predicting clinically meaningful cIMT elevation.

## 5. METHODS

### Cohort descriptions

This section describes the cohorts contributing to the cIMT EWAS and omics-based functional analyses. For the cIMT EWAS, individual-level data from the Asian cohort (HELIOS) was analysed *de novo*, while summary-level statistics from three European cohorts were incorporated. European data were obtained either from: (1) pre-existing summary statistics generated for a published meta-analysis of cIMT (Study of Health in Pomerania [SHIP]; MRC National Survey of Health and Development [NSHD], provided upon direct request from the corresponding author with permissions from the respective study teams, or (2) custom analysis conducted specifically for this investigation (Lothian Birth Cohort 1936 [LBC1936]). Detailed descriptions of the European cohorts are provided in Additional File 3. For all cohorts, participants provided written informed consent for research use of blood samples was obtained from all participants, and study protocols were approved by the relevant institutional review boards.

#### Health for Life in Singapore Study

HELIOS is a Singapore-based population cohort study designed to elucidate chronic disease determinants, enrolling 10,004 adults aged 30–84 years between 2008 and 2012, from the three major ethnic groups (Chinese, Malay and Indian).(18) Participants underwent comprehensive baseline assessments including clinical, lifestyle, and imaging evaluations including 3D carotid ultrasound, alongside biospecimen collection. All participants provided written informed consent for collection of questionnaire data, physical measurements, and biospecimens, plus storage of coded data/samples for future ethics-approved research. Participation was voluntary with rights to refuse any component or withdraw without penalty, and all received signed consent copies with study team and ethics office contacts. Self-reported ethnicity, used for ethnic-specific analyses in this investigation, showed high concordance with genetically determined ancestry (Chinese: 99.1%, Malay: 82.6%, Indian: 96.9%; Additional File 3).

HELIOS provided array-based DNA methylation data (EPIC array) from whole blood for the *de novo* cIMT EWAS, alongside whole genome sequencing (WGS) and RNA sequencing data used for downstream molecular quantitative trait locus analyses (Additional File 1: Tables S1 and S2; Additional File 3).

#### cIMT measurement

Bilateral cIMT measurements were obtained in the common carotid artery using the Philips iU 22 ultrasound system following established guidelines.(4, 19) Measurements were centered 1 cm proximal to the carotid bifurcation, avoiding plaque, and were taken from the lateral and posterior angles (four measurements in total for left and right cIMT). Automated measurements were obtained by using the Philips Qlab software to analyse images, with ECG gating to time the measurement at end-diastole. Manual review of automated measurements verified that the intima-media interface was correctly identified and traced in >95% of cases. Inter-operator and inter-reader variability was consistently low (coefficient of variation <5%). cIMT_mean_ was calculated by averaging the four measurements.

#### SG10K_Health

The SG10K_Health study integrates data from five Singapore population-based adult cohorts: HELIOS, the Singapore Epidemiology of Eye Diseases (SEED) cohort, the Multi-Ethnic Cohort (MEC), the SingHealth Duke-NUS Institute of Precision Medicine (PRISM) cohort, and the TTSH Personalised Medicine Normal Controls (TTSH) cohort. (20, 21) Four cohorts (excluding HELIOS) provided WGS and whole-blood DNA methylation profiling data (EPIC array) for *de novo* meQTL analysis to identify genetic instruments for causal inference (Table S2; Additional File 3). These methylation data were used exclusively for meQTL analysis and were not included in the cIMT EWAS.

### Quantification of DNA methylation

Genomic DNA was extracted from buffy coats and bisulfite-converted using the EZ DNA methylation kit (Zymo Research). DNA methylation was quantified using the EPIC array. Bead intensity retrieval and background correction were performed using the *minfi* R package (version 1.40.0), with a detection P-value threshold of <0.01 for marker-calling.

Quality control was applied at both the marker and sample levels to all HELIOS participants with methylation data, as previously described.(18) Of the 846,604 autosomal positions assayed by the EPIC array, we excluded markers with a low call rate (<95%; N=8,882) or those targeting non-CpG sites (N=2,839). We also excluded 58 samples due to inconsistency between reported and inferred sex (N=39), duplication (N=17) or array scanning failure (N=2); no samples failed the call rate threshold of 95%. Quantile normalisation was then applied to marker intensities to reduce between-array technical variability. After quality control, 837,722 CpG sites and 2,342 samples from the HELIOS cohort remained. Of the 2,342 samples, 1,357 had cIMT measurements. A second round of call rate filtering was applied to remove markers with low call rate in this subset. The final analytical dataset comprised 833,743 markers and 1,357 samples (Chinese n=1,063, Malay n=150; Indian n=144) with both methylation and cIMT data.

For analysis, the methylation level at each probe site was summarised as beta (β) value ranging from 0 (nonmethylated) to 1 (completely methylated). β was calculated as the ratio of the methylated signal (M) to the sum of methylated and unmethylated signal (U):

$$\beta =\frac{M}{M+U+100}$$

The four SG10K_Health cohorts providing methylation data for meQTL analysis underwent quality control procedures analogous to those applied in HELIOS. For European cohorts, whole blood-derived genomic DNA was utilised for methylation analysis using either the 450K array (LBC1936) or the EPIC array (NSHD, SHIP), also with comparable quality control procedures to HELIOS (14, 22).

### Statistical analyses of omics data

#### Epigenome-wide association analysis

EWAS performed using linear regression models with natural log-transformed cIMT_mean_ as the outcome and methylation β value at each CpG site as the predictor. Models were run separately in three HELIOS ethnic strata (Chinese, Malay and Indian; Table S1) and three European cohorts (LBC1936, SHIP, NSHD). All models used broadly comparable covariates, adjusting for age, sex, smoking status, estimated white blood cell composition (Houseman-estimated proportions or measured counts), and technical covariates (control probe principal components or array factors) (Table 1).(23, 24)

#### Meta-analysis

A two-stage meta-analysis was conducted: Stage 1 combined three HELIOS ethnic strata for Asian-focused discovery, while Stage 2 combined Asian association statistics (CpGs with P<0.05 in Stage 1) with European results (meta-analysis of LBC1936, NSHD and SHIP) for trans-ancestry meta-analysis. Due to platform differences, LBC1936 (450K array) contributed only at overlapping 450K-EPIC sites.

All meta-analyses used the sample-size weighted Stouffer method (METAL). This approach was chosen over conventional inverse variance-weighted (IVW) fixed-effects meta-analysis because effect sizes were not directly comparable across cohorts. In particular, the externally analysed European cohorts differed in cIMT measurement and summarisation protocols, as well as EWAS model specification (Table 1, Additional File 3), and harmonisation was not feasible as only summary statistics were available. Under such heterogeneity, the Stouffer Z-based method offers more robust signal detection by aggregating evidence for association without assuming comparable effect scales across studies. Within-ancestry comparison between Stouffer and IVW methods based on the complete set of markers analysed across all constituent cohorts showed near-perfect agreement the Asian meta-analysis (Spearman ρ = 0.99; p<2.2E-16; 97.0% sign concordance), and weaker but substantial agreement in European meta-analysis (Spearman ρ = 0.71; p<2.2E-16; 74.4% sign concordance). Divergence was concentrated among CpGs with high effect-size heterogeneity (I^2^), where IVW estimates attenuated relative to Stouffer, consistent with greater IVW sensitivity to between-cohort heterogeneity (Additional File 2: Figure S2). These support the suitability of the Stouffer method for discovery in the presence of cross-cohort heterogeneity, and indicate the robustness of associations to the choice of meta-analytic method.

#### Expression quantitative trait methylation

Expression quantitative trait methylation (eQTM) analysis was performed in 1,201 individuals from the HELIOS cohort to identify *cis*-CpG-gene expression associations (<1Mb) (Additional File 1: Table S2).(18) This analysis was performed using linear regression models (MatrixEQTL R package, version 2.3) with gene expression as the outcome and methylation β value at each CpG site as the predictor, adjusting for age, sex, ethnicity, RNA Integrity number (RIN), Houseman-estimated proportions of six white blood cell subpopulations, and latent factors inferred via the Probabilistic Estimation of Expression Residuals (PEER). (25)

#### Methylation quantitative trait loci

Methylation quantitative trait loci (meQTL) analysis was performed *de novo* in 5,273 individuals across four SG10K_Health adult cohorts to identify *cis*-SNPs associated with sentinel CpG methylation (<1Mb), serving as instrumental variables for causal inference (Additional File 1: Table S2). This analysis was performed using linear regression models (MatrixEQTL R package, version 2.3), with methylation β value at each CpG site as the outcome and SNP dosage as the predictor, adjusting for age, sex, ethnicity, Houseman-estimated proportions of six white blood cell subpopulations and methylation array control probe PCs. Cohort-specific results were combined using using fixed effects IVW meta-analysis (METAL).

#### Expression quantitative trait loci

Genome-wide expression quantitative trait loci (eQTL) analyses was performed in 1,168 individuals from the HELIOS cohort to identify *cis*-SNP-gene expression associations (<1Mb) ( Table S2). This analysis was performed using linear regression models (MatrixEQTL R package, version 2.3), with gene expression as the outcome and SNP dosage as the predictor, adjusting for age, sex, ethnicity, RIN and the top six PEER factors.

### Causal analyses

We investigated methylation-mediated effects using the Summary data-based Mendelian Randomisation (SMR) method and Bayesian genetic colocalisation (coloc R package, coloc.abf(), version 5.2.3).(26, 27) Causal inference analyses were restricted to genetic association data obtained from Asian cohorts to enhance population validity. SMR was performed using the Wald Ratio$\left({\hat{\beta }}_{SMR}={\hat{\beta }}_{outcome}/\right.\left.{\hat{\beta }}_{exposure}\right)$, with *cis*-meQTLs (P<1E-05, F-statistic ≥ 10) obtained from the Asian SG10K_Health cohort as genetic instruments (Table 2). Outcome summary statistics were obtained from Asian GWAS of cIMT, coronary artery disease (CAD), ischemic stroke and myocardial infarction, and eQTL studies. Arterial eQTL data were included for exploratory analysis only.

Colocalisation analysis was performed on ±1 Mb windows centered on each sentinel CpG site. For each region, coloc.abf() computes the posterior probabilities of five mutually exclusive hypotheses (H0-H4) describing the local genetic architecture of two traits, of which H3 and H4 indicate genetic effects for both traits within the analysed region— either via a shared (H4) or distinct causal variants (H3). We used the default priors (p₁=p₂=1E-04, p₁₂=1E-05) and performed sensitivity analyses by varying the shared-causality prior (p_12_). We highlight CpG-outcome pairs with SMR association (P<0.05) and colocalisation support (PP.H4>0.5) as showing preliminary evidence consistent with methylation-mediated effects via a shared causal variant. Pairs with elevated PP.H3 (>0.5) were also flagged for the possibility of shared regulation diluted by cross-ancestry LD differences or by the presence of multiple causal variants per trait in the region.

### Construction and assessment of a methylation risk score for cIMT

An MRS was constructed from sentinel CpGs for a subset of HELIOS participants analysed in the EWAS of cIMT (n=1,353; excluding 4 participants aged >80 years for whom normative ranges were not available). Participants were classified as having elevated cIMT if their cIMT_mean_ exceeded the age- sex- and ethnicity-specific 75^th^ percentile based on Asian normative ranges.(28) The HELIOS subset was split 70/30 into training (n=948) and testing (n=405) sets, stratified by ethnicity and cIMT to maintain balance. The MRS was defined as:

$$\text{MRS}=\sum_{i=1}^{k}w_{i}b_{i}$$

where k is the number of sentinel CpGs; w_i_ is the coefficient obtained from regressing sentinel CpG methylation against log-transformed cIMT_mean_ (i.e., lncIMT_mean_ ~ CpG + Age + Sex; ethnic-stratified regression followed by sample size-weighted Stouffer meta-analysis); and b_i_ is the quantile-normalised methylation beta value. Performance in the test set (n=405) was assessed using Receiver Operating Characteristic (ROC) analysis and quartile-based logistic regression across nested models:

- Model 1 (M1): cIMT_elevated_ ~ MRS + Age + Sex + Ethnicity
- Model 2 (M2): cIMT_elevated_ ~ Age + Sex + Ethnicity
- Model 3 (M3) M2 + smoking

Area Under Curve (AUC) differences were tested using DeLong’s method. For quartile analysis, potential effect modification by ethnicity was evaluated by adding an MRSxEthnicity interaction term and comparison with the main-effects model via a likelihood ratio test. Robustness was assessed by re-deriving the MRS using weights from comprehensively-adjusted models (i.e., lncIMT_mean_ ~ CpG + Age + sex + smoking status (never/current/former)+ cell composition (estimated) + PC1–30_control-probes_), matching the EWAS model specification.

### Gene set and transcription factor binding site enrichment analysis

Genes mapped to cIMT-linked CpGs were tested for overrepresentation in Gene Ontology (GO) biological processes and KEGG/REACTOME pathways, restricted to CpG-gene pairs with *cis*-eQTM support (P<0.05). In parallel, cIMT-associated CpGs were assessed for enrichment across 1,210 transcription factor binding sites (TFBS). Gene annotations were obtained from org.Hs.eg.db (v3.18.0); gene sets from msigdbr (v7.5.1); and TFBS data from ReMap 2022 (29, 30).

To address sampling bias on the EPIC array, which preferentially measures CpGs near well-annotated genes and regulatory regions with distinct methylation characteristics from CpGs in other genomic regions, a permutation-based background matching strategy was used.(31, 32) An adaptive sliding-window approach was applied to select background CpGs which were located >5-kb distance away from each cIMT-associated CpG, with gradually expanding matching thresholds (from ±0.025 to ±0.25 for mean methylation *β*-value; and ±0.0025 to ±0.025 for standard deviation), until 200 matches were found for each CpG. Enrichment was determined using a threshold of P<0.005 (i.e., none of the 200 backgrounds sets equalling or exceeding test set overlap).

## 6. RESULTS

### Study design

**Figure 1:**
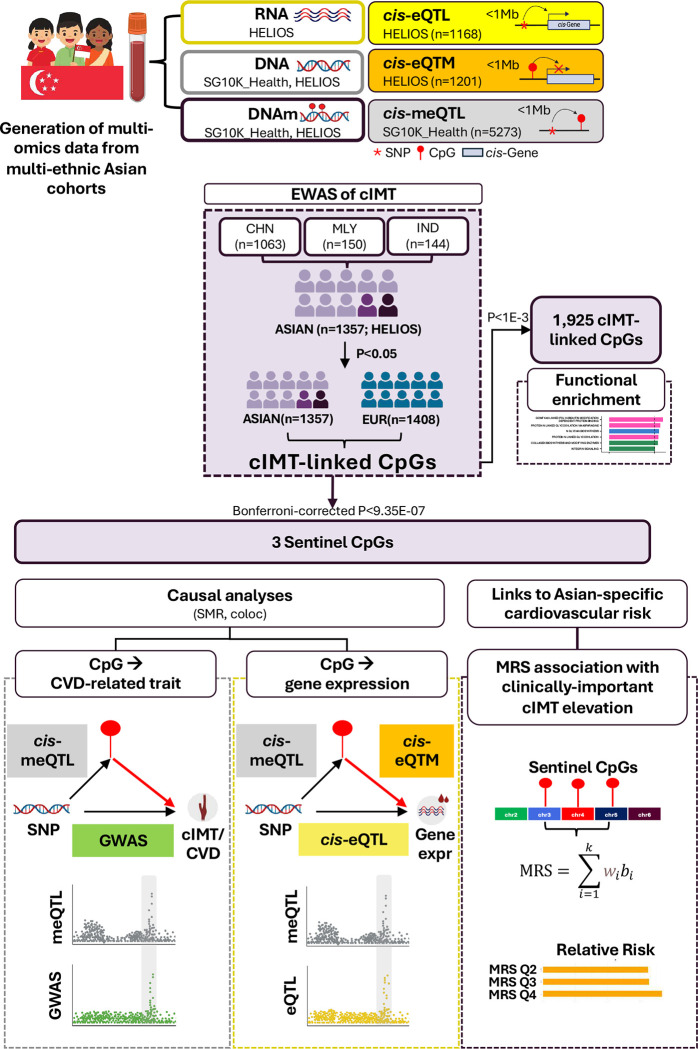
Study design. The analytical workflow integrates Asian-focused discovery with trans-ancestry validation. The Asian-focused screening stage conducted meta-analysis across ~850,000 CpG sites across three major Asian ethnic groups—Chinese, Malay, and Indian participants—from the HELIOS cohort (n=1,357). Signals reaching P<0.05 were advanced to trans-ancestry meta-analysis with European cohorts for validation. A Bonferroni-corrected significance threshold (P<9.35E-07, 0.05/53,466 CpGs tested in trans-ancestry stage) was applied to select three robust associations (‘Sentinel CpGs’) for targeted interrogation through multi-omics integration (meQTL, eQTL, eQTM analyses) and causal inference methods to examine evidence for methylation-mediated effects on cIMT, CVD and gene expression. An MRS was constructed from the three validated methylation markers and evaluated for its association with cIMT elevation above the 75^th^ percentile based on Asian normative ranges. Abbreviations: CHN, Chinese; cIMT, carotid intima-media thickness; DNAm, DNA methylation; eQTL, expression quantitative trait loci analysis; eQTM, expression quantitative trait methylation analysis; IND, Indian; meQTL, methylation quantitative trait loci analysis; MLY, Malay; MRS, methylation risk score.

Figure 1 outlines the analytical workflow. Briefly, Asian-focused screening followed by trans-ancestry meta-analysis with European cohorts was conducted to identify cIMT-associated CpG sites. A tiered inferential strategy was adopted: a suggestive threshold (P<1E-03) was applied to identify an inclusive set of CpGs for functional enrichment analyses, while a subset Bonferroni-corrected threshold (P<9.35E-07) was applied to designate independent, robust associations (‘Sentinel CpGs’) for targeted multi-omics and causal inference analyses, including SMR and colocalisation. We triangulated evidence from these analyses to prioritise sentinel CpGs with putative methylation-mediated roles in cIMT, CVD and proximal gene expression (<1Mb between CpG and gene). Finally, sentinel CpG sites were aggregated into an MRS and evaluated against population-tailored thresholds informative for increased cardiovascular risk.

### Epigenome-wide association study of carotid intima-media thickness

We identified CpG sites with cIMT-associated methylation changes using EWAS implemented within a two-stage meta-analytical framework. In Stage 1, we meta-analysed three distinct Asian ethnic strata (Chinese, Malay and Indian) from the HELIOS cohort to prioritise Asian-relevant signals, with 54,612 CpGs reaching P<0.05. (Additional File 1: Table S1, Additional File 2: Figure S1). No CpGs reached Bonferroni-corrected significance (P<0.05/833,743 markers tested) in Stage 1. Given this, we carried forward CpG sites reaching P<0.05 as a permissive initial filtering strategy to reduce the multiple testing burden in Stage 2, where a stringent Bonferroni-corrected threshold was applied .

**Figure 2:**
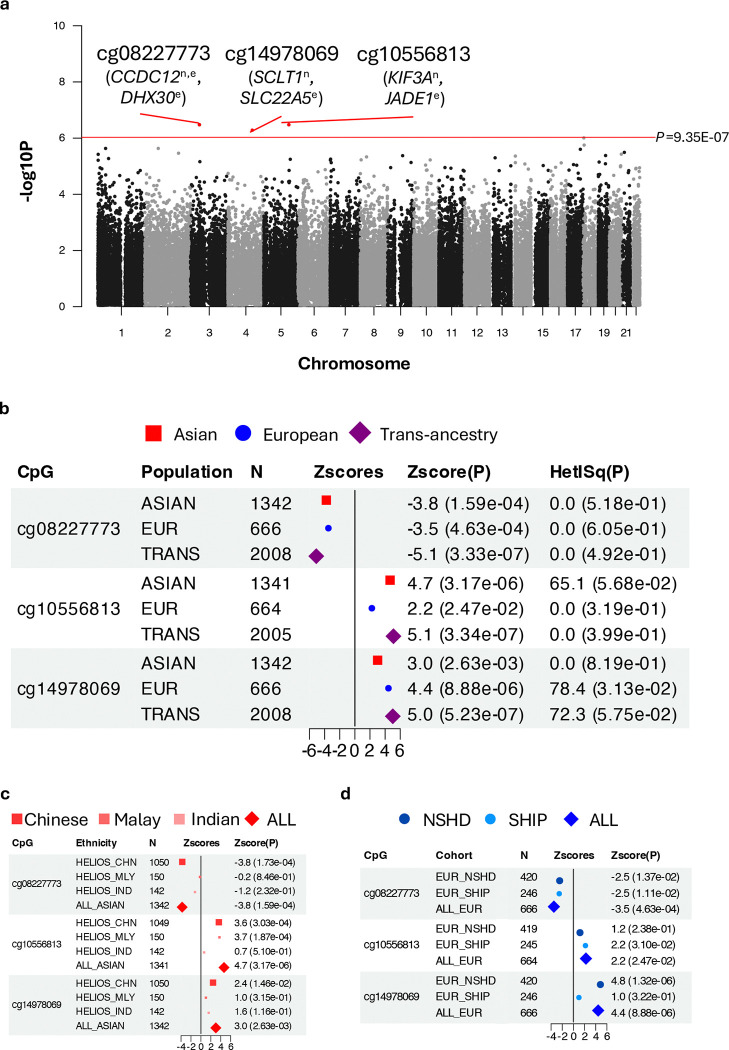
Associations between sentinel CpG sites and cIMT. **(a) Manhattan plot of cIMT associations.** Associations for the 53,466 CpGs included in the targeted trans-ancestry meta-analysis are shown. Sentinel CpGs (Bonferroni-corrected P<9.35E-07) are highlighted and annotated with proximal: genes: nearest gene (n) and top *cis*-eQTM-associated gene (e; P_eQTM_ <0.05). **(b-d) Forest plots of sentinel CpG associations in (b) trans-ancestry, (c) Asian-specific, and (d) European-specific meta-analyses, respectively.** For the European-specific meta-analysis, LBC1936 cohort (measured on 450K array) was not included since all sentinel CpGs were unique to the EPIC array. Data point sizes correspond to marker-specific effective sample sizes that are normalised to sum to one across all analysed groups. Test statistics from Stouffer’s sample-size weighted meta-analysis approach (Zscores, P) are indicated. Heterogeneity between meta-analysed groups was assessed using Cochran’s Q test and quantified by I^2^ (HetISq), with p-values indicating significance. Abbreviations: cIMT, carotid intima-media thickness; eQTM, expression quantitative trait methylation.

Methylation data were available for 53,466 of the 54,612 CpG sites (97.9%) reaching P<0.05 in Stage 1 across all Asian and European datasets analysed on the EPIC array, and these were included in the trans-ancestry meta-analysis (Figure 2a). Three CpGs were confirmed at a subset Bonferroni-corrected significance threshold of P<9.35E-07 (0.05/53,466 tests): cg08227773 (P=3.33E-07; chr3, *CCDC12* intron), cg10556813 (P=3.34E-07; chr5, *KIF3A* intron) and cg14978069 (P=5.23E-07; chr4, *SCLT1* intron) (Figure 2a-d). Since these CpGs are located on different chromosomes, they represent independent association signals, hereafter referred to as ‘sentinel CpGs’. Sentinel CpG associations persisted upon adjustment for ethnic-specific genetic principal components (Bonferroni-corrected P<0.05/3), indicating robustness to population substructure (Additional File 1: Table S3). Additionally, all sentinel CpG associations retained directional consistency in a subset of participants (n=837 out of 1,357) who did not have diagnosed diabetes, hypertension, hyperlipidaemia, were not on medication for these conditions, and showed no evidence of metabolic syndrome. (Additional File 1: Table S4; Additional File 3).

Sentinel CpGs were annotated by proximity (nearest gene) and lead *cis*-eQTM association (n=1,168, HELIOS, <1Mb). Nearest genes implicate roles in splicing (*CCDC12;* Coiled-Coil Domain Containing 12) and ciliary function (*KIF3A*, Kinesin Family Member 3A; *SCLT1*, Sodium Channel And Clathrin Linker 1).(33, 34, 35) Of these, only cg0822773-*CCDC12* was further supported by detectable *cis*-eQTM association at P<0.05 (beta=−0.84, P=6.60E-03), while *SCLT1* and *KIF3A* lacked detectable *cis*-eQTM association with their respective sentinel CpGs: (cg14978069-*SCLT1*: beta=−0.19, P=2.21E-01; cg10556813-*KIF3A* not assessed due to low expression in the dataset). Lead *cis-*eQTM genes for sentinel CpGs are involved in cellular protein synthesis (*DHX30*; DExH-Box Helicase 30), cell cycle regulation (*JADE1*; Jade Family PHD Finger 1) and fatty acid oxidation (*SLC22A5*; Solute Carrier Family 22 Member 5). *DHX30* (cg08227773-*DHX30*, beta_eQTM_=−0.84, P_eQTM_=2.05E-05) is an ATP-dependent RNA helicase that coordinates ribosome biogenesis, protein synthesis and mitochondrial metabolism and has been implicated in cancer cell survival and neurodegenerative disease. (36, 37) *JADE1* (Jade Family PHD Finger 1; cg14978069-*JADE1*, beta_eQTM_=−0.26, P_eQTM_=4.82E-02) encodes a chromatin remodeler that regulates the cell cycle, with dysregulation linked to tumorigenesis and impaired cellular proliferation or differentiation.(38, 39) *SLC22A5* (Solute Carrier Family 22 Member 5; cg10556813-*SLC22A5*, beta_eQTM_ =−0.25, P_eQTM_=6.09E-03) encodes a carnitine transporter; its expression is cytokine-sensitive and induced in monocyte differentiation, supporting metabolic control of immune function via carnitine dependent fatty acid oxidation.(40, 41)

Querying the EWAS Catalog for prior CVD/cardiometabolic associations (P<1E-04) identified links between cg08227773 and cg14978069 and incident chronic obstructive pulmonary disease, plus cg14978069 with the inflammatory marker high-sensitivity C-reactive protein; complementary GWAS Catalog lookups of proximal genes (nearest gene and lead *cis*-eQTM) revealed that several of these loci harbor variants previously associated (P<1E-05) with multiple cardiometabolic traits, inflammatory markers, and atopic conditions (asthma, eczema), collectively implicating these sentinel CpGs and their genes in inflammation, which is central to arterial remodeling (Additional File 1: Tables S5–S6).

### Enhanced discovery through inclusion of Asian cohorts

The inclusion of an Asian cohort expanded the pool of candidate signals in the current EWAS. Across 53,466 CpGs analysed in the trans-ancestry meta-analysis, Asian-European concordance of test statistics was weak (Spearman ρ=0.11, p<2.2E-16), and 48,672/53,466 CpGs (91.0%) showed detectable association (P<0.05) only in the Asian-focused screen, without corresponding evidence in the European-specific meta-analysis (P>0.05). This pattern is consistent with discovery gains attributable to the addition of an Asian cohort, and also emphasises the importance of rigorous multiplicity control to focus on the most stable signals.

**Figure 3:**
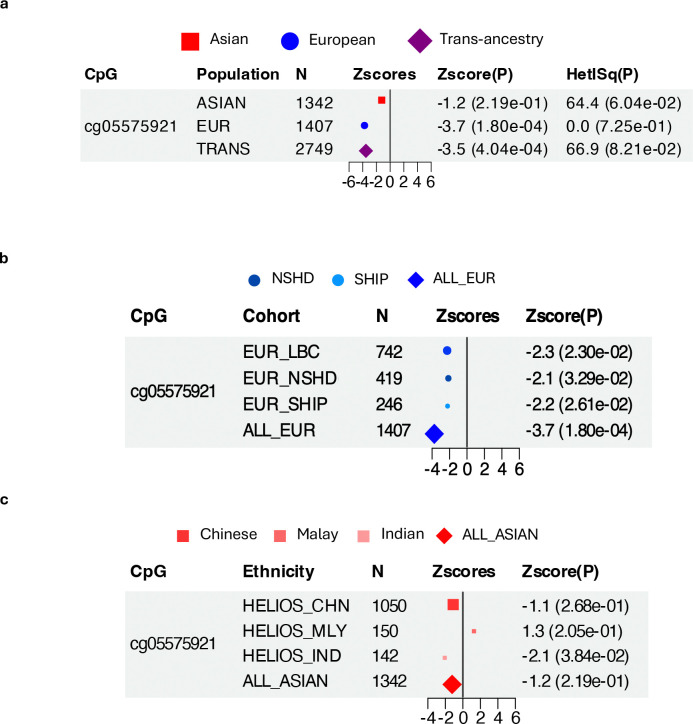
Association between cg05575921 and cIMT. The cg05575921-cIMT association was previously reported at epigenome-wide significance in a meta-analysis of European cohorts (n=6,400, Portilla-Fernández et al., 2021). **(a-c) Forest plots of the cg05575921-cIMT association** in the **(a)** trans-ancestry meta-analysis, **(b)** Asian-specific analysis and **(c)** European-specific analysis from the current investigation. The association reached P<0.05 only in the European-specific analysis, but not in the Asian-specific analysis. Data point sizes correspond to normalised effective sample size. Abbreviations: cIMT, carotid intima-media thickness.

To assess the generalisability of the most robust previously reported cIMT association— cg05575921 (chr3, *AHRR* intron), which reached epigenome-wide significance in a European-only meta-analysis (discovery n=6,400; β=−0.026; P=3.5E-08)— we performed a targeted lookup at this locus using ancestry-stratified and trans-ancestry meta-analysis. Although the association was present and directionally-consistent in the trans-ancestry meta-analysis (Z_trans_ =−3.5, P=4.04E-04) (Figure 3a), it was driven by Europeans (Z=−3.7, P=1.80E-04) (Figures 3a,b), with attenuated effect in Asians (Z=−1.2, P=2.19E-01) (Figures 3a,c), possibly reflecting differences in exposure distributions.

### Gene set and transcription factor binding site overrepresentation analysis

**Figure 4:**
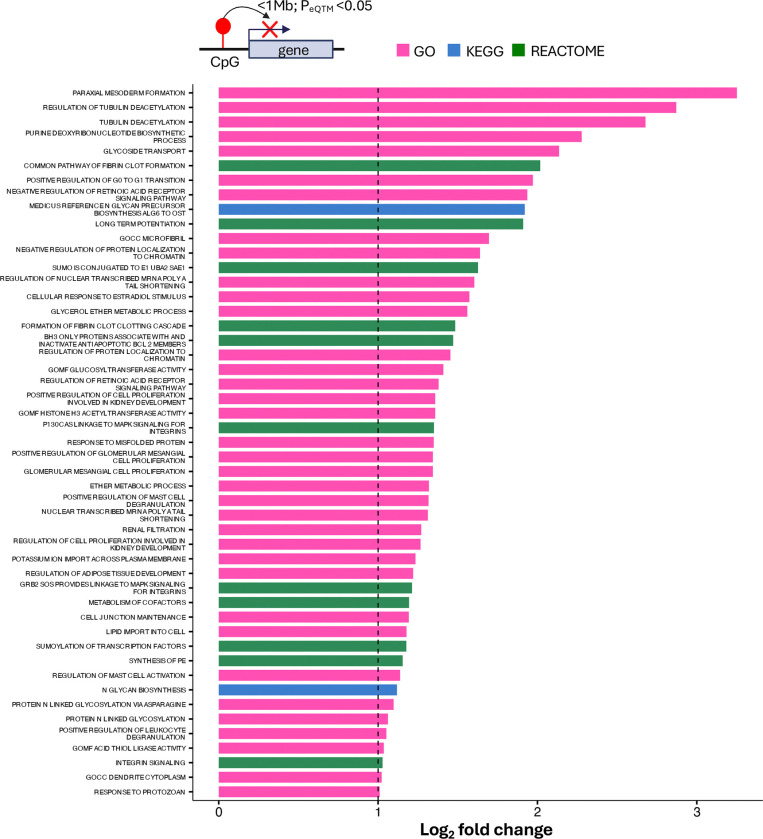
Overrepresented gene sets among genes mapped to cIMT-linked CpGs. Genes mapped to cIMT-linked CpGs were analysed for pathway enrichment against a background of genes mapped to 200 permutations of EPIC array CpGs of matched methylation level and variability to the cIMT-linked CpGs. CpG-gene mapping was done via *cis*-eQTM analysis (P_eQTM_<0.05). Enriched gene sets (P<0.005, log_2_ fold-change >1) are displayed.

To identify biological pathways potentially affected by cIMT-associated methylation changes, we performed gene set overrepresentation analysis on an inclusive set of 1,925 CpGs (P<1E-03). Of the 1,925 CpGs, *cis*-eQTM relationships (P<0.05) were identified between 1,298 CpGs and 3,337 unique genes (<1Mb). For enrichment testing, these 3,337 genes served as the test set, compared against a background derived from 200 permuted sets of EPIC array CpGs matched to the 1,925 CpGs for methylation levels and variability. Together, the blood-enriched terms suggest a systematic arterial remodeling program encompassing re-activation of developmental circuits, immune activation, as well as metabolic and cytoskeletal rewiring which are known to modulate leukocyte adhesion, endothelial barrier function and mechanosensing. (Figure 4)

To further explore potential regulatory mechanisms, we tested the 1,925 cIMT-linked CpGs for enrichment in TFBS compared to matched background EPIC array CpGs. cIMT-linked CpGs were enriched for binding sites of 63 transcription factors (P <0.005), including *MEF2C* (top hit; fold-change=2.10 relative to background mean; P <0.005) and others with established roles in vascular remodeling (Additional File 1: Table S7). Endothelial *MEF2C* deficiency disrupts atheroprotective mechanisms, increases inflammatory activation and leads to rapid vascular dysfunction *in vivo*, likely through NF-κB signaling. (42, 43) However, Mef2c regulates macrophage polarization independently of NF-κB signaling in mice, indicating coordinate, context-dependent contributions of *MEF2C* to arterial remodeling across different cell types. (44)

### Genetically-anchored causal inference analyses to explore sentinel CpG-mediated pathways

Beyond observational associations, genetically-anchored causal inference analyses were applied to evaluate sentinel CpG-mediated pathways to cIMT and atherosclerotic CVD (CAD, ischemic stroke and myocardial infarction); and to putative effector genes (Table 2). Causal inference analyses were restricted to genetic association inputs from Asian cohorts to enhance population validity.

**Figure 5:**
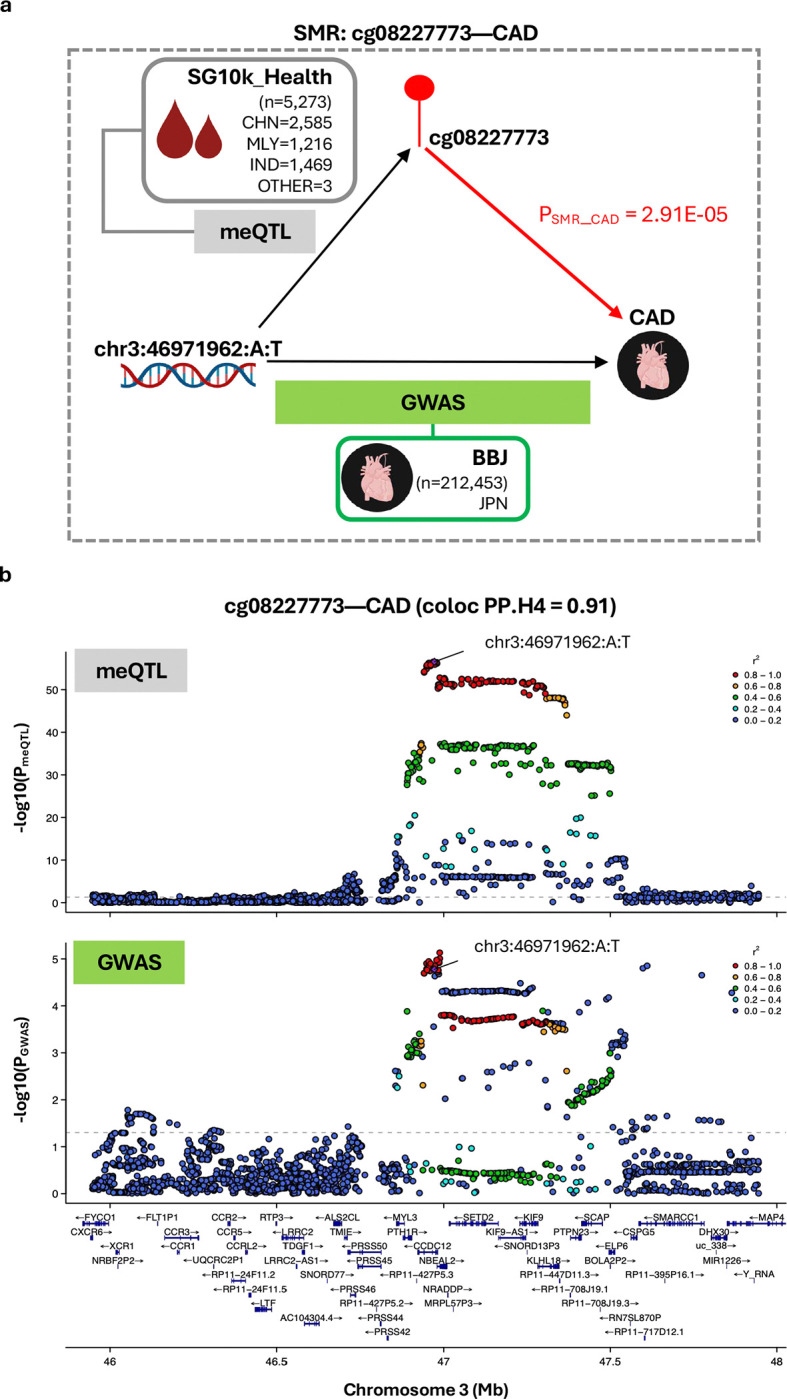
Evidence for cg08227773 methylation-mediated effects on CAD risk. **(a) Schematic overview of the SMR experiment.** *cis*-meQTL data (exposure) were derived from Asian cohorts in SG10K_Health (n=5,273). CAD GWAS summary statistics (outcome) were obtained from Biobank Japan (n=212,453 including 29,139 cases and 183,134 controls). The instrumental SNP is chr3:46971962:A:T (P_meQTL_=2.79E-57, F-statistic=306.25). **(b) Regional association plots displaying genetic associations for cg08227773 methylation and CAD.** Plots display cg08227773 meQTL (top) and CAD GWAS (bottom) signals within a ±1Mb window centered on cg08227773. The ±1Mb window size reflects the *cis*-region used for meQTL mapping in the SG10K_Health cohort. Colocalisation analysis supported shared causal variant (PP.H4=0.91). SNPs are colored according to their linkage disequilibrium (r^2^) with the instrumental SNP, calculated using the full SG10K_Health cohort with available genetic data (N=9,766). Gene tracks show locations of protein-coding genes based on Ensembl Release 86 (hg38). Abbreviations: BBJ, Biobank Japan; CAD, coronary artery disease; CHN, Chinese; IND, Indian; JPN, Japanese; MLY, Malay; meQTL, methylation quantitative trait loci analysis.

Of all CpG-trait pairs evaluated, evidence compatible with methylation-mediated involvement was observed only for cg08227773-CAD (P_SMR_ = 2.91E-05), with colocalisation indicating a 91% posterior probability that altered cg08227773 and CAD risk are driven by a shared genetic variant (coloc PP.H4=0.91) (Figure 5; Additional File 1: Table S8). We tested how sensitive the colocalisation inference was to the assumed chance that the same variant affects both traits (p_12_). Across a wide range of plausible values (2.51E-06 to 3.16E-05, including the default 1E-05), the H4 call remained robust (coloc PP.H4>0.5 and H4/H3 ≥3), indicating stable shared-signal inference. To minimise LD mismatches from combining a multi-ethnic meQTL dataset with an East Asian-only GWAS, we repeated colocalisation analysis using *cis*-meQTL effects from the Chinese-only subset of the SG10K_Health meQTL cohort (n=2,585). Across 3,107 variants common to both datasets, the posterior for a shared causal variant remained high (PP.H4=0.89), suggesting the colocalisation is unlikely reflect artefacts from ancestry-related LD differences.

**Figure 6:**
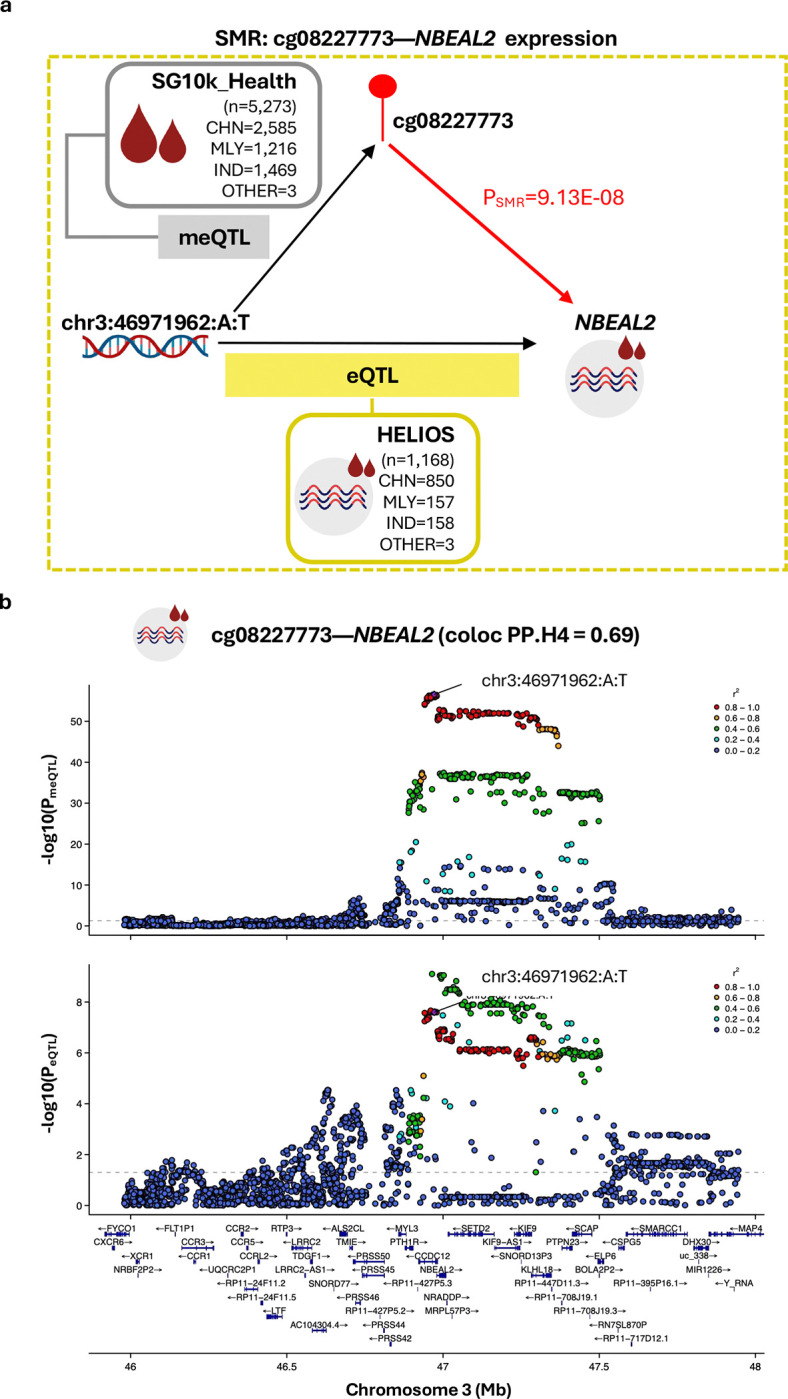
Evidence for cg08227773 methylation-mediated effects on *NBEAL2* expression. **(A) Schematic overview of the SMR experiment.** *cis*-meQTL data (exposure) were derived from Asian cohorts in SG10K_Health (n=5,273). Blood-based *cis*-eQTL data (outcome) were obtained from the Asian HELIOS cohort (n=1,168) for primary analyses. The instrumental SNP for SMR is chr3:46971962:A:T (P_meQTL_=2.79E-57, F-statistic=306.25). **(B) Regional association plots displaying genetic associations for cg08227773 methylation and *NBEAL2* expression.** Plots display cg08227773 meQTL (top) and *NBEAL2* eQTL (bottom) signals within a ±1Mb window centered on cg0822773. The ±1Mb window size reflects the *cis*-region used for meQTL mapping in the SG10K_Health cohort. Colocalisation analysis supported a shared causal variant (PP.H4=0.69). SNPs are colored according to their linkage disequilibrium (r^2^) with the instrumental SNP, calculated using the full SG10K_Health cohort with available genetic data (N=9,766). Gene tracks show locations of protein-coding genes based on Ensembl Release 86 (hg38). Abbreviations: CHN, Chinese; IND, Indian; MLY, Malay.

Causal analyses with proximal gene expression as outcome were performed between two sentinel CpGs and 26 unique proximal genes (±1Mb) expressed in our whole blood RNAseq dataset (HELIOS, n=1,168) with suitable genetic instruments identified. Of these, evidence compatible with methylation-mediated involvement was observed only for cg08227773-*NBEAL2* (P_SMR_=9.13E-08), with colocalisation indicating a 69% posterior probability that altered cg08227773 methylation and *NBEAL2* expression are driven by a shared genetic variant (Figure 6; Additional File 1: Table S9). Sensitivity analyses across plausible sharing priors showed that the H4 call remained robust over a wide range of values (1.58E-05 to 3.16E-05, including the default 1E-05), indicating a stable shared-signal inference. SMR analyses using arterial eQTL data suggest that the blood-supported cg08227773-*NBEAL2* relationship may generalise to arterial contexts (aorta P_SMR_=1.75E-04; coronary artery P_SMR_=1.36E-04;, and tibial artery P_SMR_= 2.29E-12; each instrumented by the same *cis-*variant at chr3:46971962:A:T), although interpretation remains preliminary given the mismatch in both ancestral and tissue contexts of the genetic association data (Asian blood-based meQTL and European arterial eQTL; Table 2) which may reduce power and introduce bias. Mechanistically, *NBEAL2* (Neurobeachin-like 2) is required for platelet α-granule biogenesis; loss-of function mutations in *NBEAL2* causes Grey Platelet Syndrome, an inherited bleeding disorder (45, 46, 47). *NBEAL2* deficiency is also linked to immune dysregulation, characterised by heightened expression of activation and degranulation markers on neutrophils and eosinophils, as well as elevated proportions of specific adaptive immune lymphocytes.(48) Together, these findings support biological plausibility for a cg08227773–*NBEAL2* regulatory axis in arterial remodeling.

Apart from cg08227773-*NBEAL2,* causal inference analyses further identified 16 CpG-gene pairs for which the analysed genomic window shows effects for both methylation and expression, but likely driven by distinct variants (P_SMR___expr_ <0.05, coloc PP.H3 >0.5) (Additional File 1: Table S9). While high PP.H3 formally indicates distinct variants under coloc’s single-variant model, elevated PP.H3 can also arise under truly shared-variant scenarios— particularly in multi-ethnic analyses, where cross-dataset LD differences reduce concordance, or when multiple causal variants per trait dilute an apparent single-variant colocalization signal. These 16 pairs, prioritised for future fine-mapping to resolve locus architecture, implicate genes relevant to arterial remodeling across leukocyte trafficking, inflammation and platelet function, as well as broader roles in cytoskeletal dynamics, mechanosensing and transcriptional control (Additional File 1: Table S9).

### Development and validation of a methylation risk score for clinically meaningful risk elevation

To assess clinical relevance of the methylation perturbations, we combined the three sentinel CpGs into a weighted MRS evaluated its relationship with clinically meaningful cIMT elevation—defined as cIMT at or above the age-, sex- and ethnicity-specific 75^th^ percentile, a threshold defined by the 2008 American Society of Echocardiography guidelines and reinforced by contemporary evidence of higher CVD event rates amongst individuals at or above this threshold (4, 49). We used a 70:30 train-test split in the HELIOS cohort (N=1,353), stratified by ethnicity and the prevalence of at-risk cIMT, to fit the MRS in the training set (n=948) and evaluate performance in the testing set (n=405). The MRS was constructed using CpG weights estimated in models adjusted for age, sex, and ethnicity; covariates were limited to these basics to mitigate overfitting given the small sample size.

**Figure 7:**
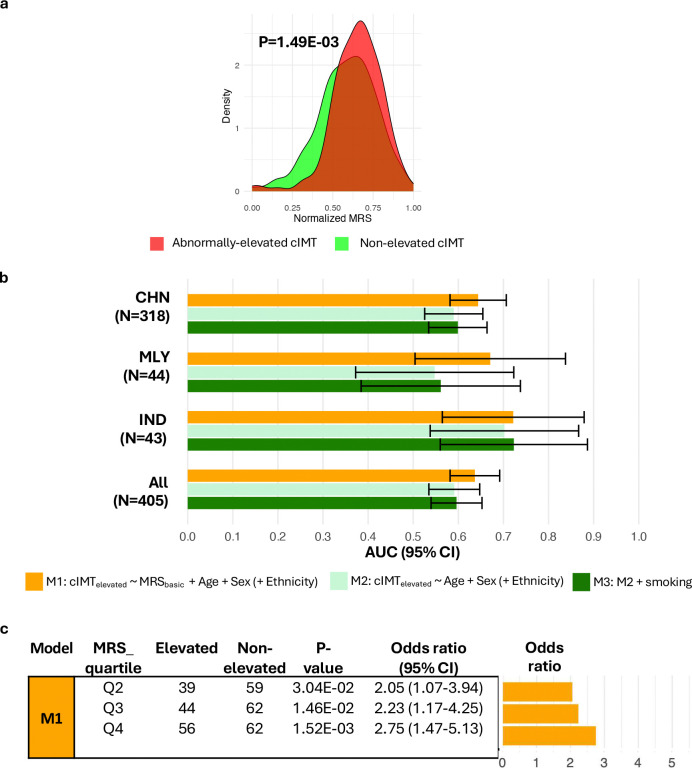
MRS of cIMT predicts clinically meaningful cIMT elevation. The MRS was built from sentinel CpGs, weighting each CpG by its Stouffer-combined z-score estimated in the training set (model: log-transformed cIMT_mean_ ~sentinel CpG methylation + age + sex). Models were fit separately by ethnicity, and the per-ethnicity association z-scores were combined using Stouffer’s method to obtain the final CpG weight. **(a) Distribution of MRS by cIMT status.** MRS values were normalised within each group for visualisation purposes. P-values are from two-sample t-tests using the original (unnormalised) scores. **(b) Discriminatory ability of MRS.** ROC analysis compares models with and without the MRS; AUCs are plotted **(c) Stratification by MRS quartiles.** Participants were grouped by MRS quartile; relative odds of clinically concerning cIMT elevation are reported relative to Q1 (lowest MRS). Odds ratios and 95% CI were obtained from logistic regression (cIMT_elevated_ ~ MRS quartile + Age + Sex + Ethnicity) by exponentiating the MRS quartile coefficients. Abbreviations: AUC, Area Under Curve; CHN, Chinese; IND, Indian; MLY, Malay; MRS, methylation risk score; ROC, Receiver Operating Characteristic.

In this Asian cohort, MRS differed between people with elevated cIMT and those with normal cIMT (P=1.49E-03, Figure 7a). Ethnic-specific MRS distributions did not differ detectably, indicating limited evidence for the influence of ethnic-related heterogeneity on the observed separation at the current sample sizes (Additional File 2: Figure S3a). Across ethnic strata, the MRS distribution was shifted toward higher values among individuals with above-threshold cIMT; however the separation only reached P<0.05 in the Chinese ethnic strata (n=318), possibly reflecting limited power in the Malay (n=44) and Indian (n=43) samples (Additional File 2: Figure S3b).

Receiver Operating Characteristic (ROC) analysis across three stepwise models (M1-M3) showed that the MRS provides moderate discrimination for above-threshold cIMT (Figure 7b; Additional File 1: Table S10). There were no detectable differences in MRS discriminatory ability (AUC for M1) across ethnic strata (pairwise DeLong tests for AUC differences: all P>0.05). In the overall multi-ethnic cohort, adding the MRS to a demographics-only baseline (M2 versus M1) increased Area Under Curve (AUC) by 7.72% (AUC=0.59 to AUC=0.64; P=2.79E-02) (Additional File 1: Table S10). These findings suggest that the MRS adds modest discriminatory signal for above-threshold cIMT.

Quartile analyses showed a graded association between MRS and above-threshold cIMT, with individuals at the highest MRS quartile (Q4) exhibiting 2.75-fold higher odds of abnormal cIMT elevation compared to the lowest quartile (95% CI: 1.47–5.13; P=1.52E-03, Figure 7c). Including an MRSxEthnicity interaction term in the logistic regression did not improve model fit (likelihood-ratio P>0.05), providing no detectable evidence that the association between MRS and elevated cIMT differs by ethnicity at current sample sizes.

To assess robustness to potential confounders, we derived an alternative score (MRS_comprehensive_) using CpG weights obtained from ethnicity-specific models adjusted for age, sex, smoking status, estimated white blood-cell composition and array control-probe PCs; the resulting weights were then meta-analysed across ethnicities, matching the EWAS specification. Compared to the basic score, MRS_comprehensive_ showed a larger between-group differences (above-threshold versus normative cIMT P=6.15E-04; Additional File 2: Figure S4a versus Figure 7a), better discrimination (fully adjusted model M3 AUC=0.75 (95% CI:0.71–0.80), Additional File 2: Figure S4b versus Figure 7b) and stronger risk stratification (Q4 versus Q1 OR=5.02, (95% CI: 2.31–10.89); Additional File 2: Figure S4c versus Figure 7c). Although comprehensive covariate adjustment strengthens the effects, these patterns are already evident with the basic score, indicating that the observed associations are robust to covariate specification.

## 7. DISCUSSION

The current EWAS meta-analysis of cIMT leveraged a multi-ethnic Asian cohort and expanded EPIC array coverage to elucidate methylation-related pathways of arterial remodeling. To capture signals from Asians, previously noted to have unique cIMT etiology but underrepresented in molecular studies, Asian-focused discovery (n=1357) was undertaken to prioritise signals before trans-ancestry meta-analysis to boost statistical power (n=2765). Three sentinel CpGs surfaced by trans-ancestry meta-analysis (subset Bonferroni P<9.35E-07) underwent in-depth characterisation using population-matched resources, including well-powered genetic association data and calibrated cIMT thresholds. Causal inference analyses leveraging some of the largest Asian genetic association studies within their respective phenotypes provided evidence compatible with cg08227773 methylation-mediated effects on coronary artery disease (CAD) risk and the expression of the immune regulator *NBEAL2* via a shared causal variant. An MRS aggregating the three sentinel CpGs showed preliminary discriminatory ability against Asian-tailored cIMT thresholds in split-sample internal validation (70% training, 30% hold-out testing), with odds of elevated cIMT tracking progressively with methylation burden. Together, these findings demonstrate that Asian cohort inclusion yields insights relevant to arterial remodelling that may extend to overt clinical CVD, providing a foundation for replication and extension in other Asian cohorts with integrated cIMT phenotyping and methylation data as such resources become available.

We acknowledge the permissive filtering threshold of P<0.05 used to carry forward candidates form the Asian meta-analysis into the trans-ancestry stage, where a stringent Bonferroni-corrected threshold was applied to mitigate the liberal discovery filter. This approach, and the lack of signal detected at Bonferroni-significance in the discovery stage, reflect the modest individual methylation effect sizes expected for cIMT, concordant with the largest prior European EWAS meta-analysis (n=6400) identifying only a single Bonferroni-significant association at cg05575921.

Our trans-ancestry datasets enabled a closer assessment of whether the sole reported cIMT-associated biomarker, cg05575921, generalises across populations. This canonical smoking biomarker showed evidence of association in the trans-ancestry analysis but not within the Asian-only subset; consistent with prior findings in Europeans, the overall signal appeared largely European-driven, with directionally consistent but statistically inconclusive evidence in the Asian-specific analysis (P>0.05). Definitive inferences about population specificity cannot be drawn due to potential non-biological sources of heterogeneity, including differences in study settings, exposure distribution, cIMT measurement protocols, phenotypic derivation and covariate specification. Importantly, because the European cohorts included in our analysis were also part of the previous meta-analysis, this does not constitute an independent replication, instead serving as an assessment of consistency and potential transferability outside the European setting.

A key contribution of this work is its application of genetically-anchored causal inference methods to explore the mediatory role of sentinel CpGs, beyond observational associations. We leveraged recent, large-scale Asian GWAS datasets for cIMT (HELIOS cohort, n=6,798) and CVD (East Asian cohorts, n=161,206–256,274), and addressed the lack of comprehensive Asian QTL resources by conducting *cis*-meQTL analysis in 5,273 multi-ethnic Asian individuals (SG10K_Health) and *cis*-eQTL analysis and in a non-overlapping cohort of 1,168 multi-ethnic Asian individuals (HELIOS). While these analyses did not suggest sentinel CpG methylation-mediated effects on cIMT— possibly limited by the relatively modest GWAS sample size— our analyses provide evidence consistent with cg08227773-mediated effects on CAD risk and *NBEAL2* expression via a shared causal variant. Beyond established roles in platelet function and thrombosis, emerging descriptions of *NBEAL2*’s immunoregulatory roles in lymphocytes and neutrophils suggest broader influences on the inflammatory milieu with potential effects on arterial remodeling warranting further exploration.(48, 50, 51) However, mechanistic refinement is required, as the single-instrument SMR analyses undertaken in this investigation preclude pleiotropy-robust approaches that require multiple LD-independent instruments, leaving causal direction suggested but not established. Furthermore, while the vascular relevance of cg08227773-*NBEAL2* is suggested by SMR analyses using arterial eQTL data, this finding should be considered hypothesis-generating given the tissue and population mismatch between the Asian blood-derived meQTL and European arterial eQTL data used in this exploratory analysis. Priorities for mechanistic refinement include: (i) repeating the causal inference analyses using expanded Asian meQTL resources with sufficient power within individual ethnic subsets to obtain robust genetic associations and to define LD-independent instruments; and (ii) experimental manipulation of cg08227773 methylation and *NBEAL2* expression in relevant vascular cell types to assess vascular relevance.

The cIMT-linked methylation alterations were also associated with Asian-tailored cIMT thresholds informative for cardiovascular risk, consistent with capture of disease-relevant biology. An MRS constructed from three sentinel CpGs differentiated risk groups and showed graded increase in odds of beyond-threshold cIMT elevation. However, this MRS analysis was limited by reliance on internal split-sample validation within the HELIOS cohort, as no external validation cohort with matched methylation and cIMT data was available. External validation in an independent cohort remains essential to establish generalisability and to assess clinical utility with greater rigor. Priorities to improve the MRS analysis include expanding training datasets to derive robust weights and validation in fully independent cohorts as suitable resources become available. To assess clinical value more definitively, MRS should also be tested for association with incident atherosclerotic CVD events and for incremental predictive performance beyond cIMT and standard risk factors, leveraging biobank-scale resources integrating multi-omics, detailed phenotypes, and longitudinal outcomes (e.g., from electronic health records.)

## 8. CONCLUSION

This study identifies three novel blood-based DNA methylation markers for cIMT using an Asian-led, trans-ancestry discovery strategy; among these, cg08227773 showed causal-inference evidence consistent with methylation-mediated effects on CAD risk and with regulation of *NBEAL2* expression. MRS aggregating these three signals associated with cIMT elevation defined using Asian-specific thresholds. These findings nominate biologically relevant targets while highlighting the need for larger, population-matched multi-omics resources and longitudinal CVD outcomes to refine mechanisms and more rigorously evaluate clinical utility.

## Supplementary Material

This is a list of supplementary files associated with this preprint. Click to download.

• 20260226additionalfile1.xlsx

• 20260226additionalfile3.docx

• 20260226additionalfile2.docx

Additional File 1(‘2026-02-26 additional_file_1.xlsx’): Cohort characteristics, extended data from core analyses and sensitivity analyses

Additional File 2(‘2026-02-26 additional_file_2.docx’): Supplementary Figures

Additional File 3(‘2026-02-26 additional_file_3.docx’): Extended cohort descriptions and methodological details.

## Figures and Tables

**Table 1.** Models for epigenome-wide association study of cIMT across cohorts

Cohort	Ethnic strata	Covariates	Array
HELIOS (n=1,357)	Chinese (n=1063)	Age + sex + smoking status (never/current/former)+cell composition (estimated) + PC1–30_control-probes_	EPIC
	Malay (n=150)		
	Indian (n=144)		
LBC1936 (n=742)	NA	Age + sex + smoking status (current vs ex/never) + cell counts + PC1–30_control-probes_	450K
NSHD (n=420)	NA	Age + sex + smoking status (never/current/former) + cell counts + array ID + array position	EPIC
SHIP (n=246)	NA	Age + sex + smoking status (never/current/former) + cell counts + PC1–6_cell_composition_	EPIC

All models used natural log-transformed cIMT_mean_ as the outcome. In HELIOS, EWAS analyses was performed within each ethnic stratum (Chinese, Malay, Indian) using identical covariate models; strata-specific results were then combined through fixed-effects meta-analysis to obtain overall Asian summary statistics. Technical variation was addressed using either control probe principal components (PC1–30_control-probes_) or array-level factors (array ID and position). Cell composition was adjusted using estimated white blood cell proportions derived from methylation data via the Houseman reference-based deconvolution method or direct cell counts.

**Table 2:** Data sources for Summary data-based Mendelian Randomisation and colocalisation analyses

Exposure		Exposure population (ancestry/ethnic group; N)		Outcome		Outcome data source		Outcome population (ancestry/ethnic group; N)	
SG10K_Health (meQTL)		Southeast Asian: Chinese, Malay, Indian, Other ethnic groups (N=5,273)		cIMT		HELIOS		Southeast Asian: Chinese, Malay, Indian ethnic groups; N=6,798	
				Coronary artery disease		Biobank Japan (52)		East Asian: Japanese; N=212,453 (Cases=29,319; Controls=183,134)	
				Ischemic Stroke		Mishra et al. (53)		East Asian: Japanese, Korean, Chinese; N=256,274 (Cases=19,032; Controls=237,242)	
				Myocardial Infarction		Biobank Japan (54)		East Asian: Japanese; N=161,206 (Cases=14,992; Controls=146,214)	
				Gene expression (blood)		HELIOS		Southeast Asian: Chinese, Malay Indian, Other ethnic groups; N=1,168	
Gene expression (aorta)		GTEx v10 (55)		European: N=472	
Gene expression (coronary artery)				European ancestry: N=268	
Gene expression (tibial artery)				European ancestry: N=689	

*cis*-meQTL (<1Mb) from the multi-ethnic Asian SG10K_Health cohort were used as genetic instruments. Outcome data were obtained from GWAS of cardiovascular phenotypes (cIMT in in Southeast Asian populations; coronary artery disease, ischemic stroke and myocardial infarction in East Asian populations) and eQTL analyses in blood (Southeast Asian ancestry) or arterial tissue (European ancestry). Sample sizes represent total participants, with case-control numbers specified for binary outcomes. Abbreviations: cIMT, carotid intima–media thickness; eQTL, expression quantitative trait loci analysis; GTEx, Genotype-Tissue Expression; GWAS, genome-wide association study; meQTL, methylation quantitative trait loci analysis; SMR, Summary-data Mendelian randomisation.

## Data Availability

The datasets supporting the conclusions of this article are included within the article and its additional files. Scripts used for the analysis are available at: https://github.com/KonstanzeTan/cIMT-EWAS. Additional code is available from the authors upon reasonable request.

## The HELIOS Study Team

Akash Bahai^1^, Benjamin Lam^2,3^, Dorrain Low^1^, Elio Riboli^4^, Gervais Wansaicheong^1,5^, Harinakshi Sanikini^3^, Hong Kiat Ng^1^, Jimmy Lee^1,6^, Joanne Ngeow^1,7^, Kelvin Li^8^, Kostas Tsilidis^4^, Nilanjana Sadhu^1^, Paul Elliott^4^, Sabrina Wong^1,9^, Terry Tong^1^, Tock Han Lim^8^, Tricia Chang^10^, Wai Kee Kok^10^, Xiaoyan Wang^1^, Yik Weng Yew^1,11^

1. Population and Global Health, Nanyang Technological University, Lee Kong Chian School of Medicine, Singapore, Singapore.

2. Nanyang Technological University, Lee Kong Chian School of Medicine, Singapore, Singapore.

3. Khoo Teck Puat Hospital, Singapore, Singapore.

4. Department of Epidemiology and Biostatistics, Imperial College London, School of Public Health, London, United Kingdom.

5. Diagnostic Radiology, Tan Tock Seng Hospital, Singapore, Singapore.

6. Research Division, Institute of Mental Health, Singapore, Singapore.

7. Division of Medical Oncology, National Cancer Centre, Singapore, Singapore.

8. Ophthalmology, Tan Tock Seng Hospital, Singapore, Singapore.

9. Clinical Research Unit, National Healthcare Group Polyclinics, Singapore, Singapore.

10. National Healthcare Group Polyclinics, Singapore, Singapore.

11. Research Division, National Skin Centre, Singapore, Singapore.

Certain figures in this publication were created using BioRender (https://BioRender.com/0q97c0o).

## 9. LIST OF ABBREVIATIONS

450K array: Illumina Infinium HumanMethylation450 BeadChip
CAD: Coronary artery disease
cIMT: Carotid intima-media thickness
CpG: Cytosine-phosphate-Guanine dinucleotide, referring to primary sites of DNA methylation in the genome where cytosine is followed by guanine
CVD: Cardiovascular disease
EPIC array: Illumina Infinium HumanMethylationEPIC BeadChip
eQTL: Expression quantitative trait locus (SNP—gene expression association)
eQTM: Expression quantitative trait methylation (association between CpG methylation and gene expression)
EWAS: Epigenome-wide association analysis
GWAS: Genome-wide association study
LD: Linkage disequilibrium
meQTL: Methylation quantitative trait locus (SNP—CpG methylation association)
MRS: Methylation risk score
PC: Principal components
PCA: Principal component analysis
SMR: Summary data-based Mendelian Randomisation
SNP: Single nucleotide polymorphism
TFBS: Transcription factor binding sites

## References

[1] Global Burden of Cardiovascular D, Risks C. Global, Regional, and National Burden of Cardiovascular Diseases and Risk Factors in 204 Countries and Territories, 1990–2023. J Am Coll Cardiol. 2025. 10.1016/j.jacc.2025.08.015 40990886

[2] Berenson GS, Srinivasan SR, Bao W, Newman WP 3rd, Tracy RE, Wattigney WA. Association between multiple cardiovascular risk factors and atherosclerosis in children and young adults. The Bogalusa Heart Study. N Engl J Med. 1998;338(23):1650–6. 9614255 10.1056/NEJM199806043382302

[3] Touboul PJ, Hennerici MG, Meairs S, Adams H, Amarenco P, Bornstein N, Mannheim carotid intima-media thickness and plaque consensus (2004–2006-2011). An update on behalf of the advisory board of the 3rd, 4th and 5th watching the risk symposia, at the 13th, 15th and 20th European Stroke Conferences, Mannheim, Germany, 2004, Brussels, Belgium, 2006, and Hamburg, Germany, 2011. Cerebrovasc Dis. 2012;34(4):290–6. 23128470 10.1159/000343145 PMC3760791

[4] Stein JH, Korcarz CE, Hurst RT, Lonn E, Kendall CB, Mohler ER, Use of carotid ultrasound to identify subclinical vascular disease and evaluate cardiovascular disease risk: a consensus statement from the American Society of Echocardiography Carotid Intima-Media Thickness Task Force. Endorsed by the Society for Vascular Medicine. J Am Soc Echocardiogr. 2008;21(2):93–111; quiz 89–90. 18261694 10.1016/j.echo.2007.11.011

[5] Pignoli P, Tremoli E, Poli A, Oreste P, Paoletti R. Intimal plus medial thickness of the arterial wall: a direct measurement with ultrasound imaging. Circulation. 1986;74(6):1399–406. 3536154 10.1161/01.cir.74.6.1399

[6] Bots ML. Carotid intima-media thickness as a surrogate marker for cardiovascular disease in intervention studies. Curr Med Res Opin. 2006;22(11):2181–90. 17076979 10.1185/030079906X148472

[7] Lorenz MW, Markus HS, Bots ML, Rosvall M, Sitzer M. Prediction of clinical cardiovascular events with carotid intima-media thickness: a systematic review and meta-analysis. Circulation. 2007;115(4):459–67. 17242284 10.1161/CIRCULATIONAHA.106.628875

[8] Willeit P, Tschiderer L, Allara E, Reuber K, Seekircher L, Gao L, Carotid Intima-Media Thickness Progression as Surrogate Marker for Cardiovascular Risk: Meta-Analysis of 119 Clinical Trials Involving 100 667 Patients. Circulation. 2020;142(7):621–42. 32546049 10.1161/CIRCULATIONAHA.120.046361 PMC7115957

[9] Nonterah EA, Crowther NJ, Klipstein-Grobusch K, Oduro AR, Kavousi M, Agongo G, Racial and Ethnic Differences in the Association Between Classical Cardiovascular Risk Factors and Common Carotid Intima-Media Thickness: An Individual Participant Data Meta-Analysis. J Am Heart Assoc. 2022;11(15):e023704. 10.1161/JAHA.121.023704 PMC9375511 35876421

[10] Yeung MW, Wang S, van de Vegte YJ, Borisov O, van Setten J, Snieder H, Twenty-Five Novel Loci for Carotid Intima-Media Thickness: A Genome-Wide Association Study in >45 000 Individuals and Meta-Analysis of >100 000 Individuals. Arterioscler Thromb Vasc Biol. 2022;42(4):484–501. 34852643 10.1161/ATVBAHA.121.317007 PMC8939707

[11] Franceschini N, Giambartolomei C, de Vries PS, Finan C, Bis JC, Huntley RP, GWAS and colocalization analyses implicate carotid intima-media thickness and carotid plaque loci in cardiovascular outcomes. Nat Commun. 2018;9(1):5141. 30510157 10.1038/s41467-018-07340-5 PMC6277418

[12] Strawbridge RJ, Ward J, Bailey MES, Cullen B, Ferguson A, Graham N, Carotid Intima-Media Thickness: Novel Loci, Sex-Specific Effects, and Genetic Correlations With Obesity and Glucometabolic Traits in UK Biobank. Arterioscler Thromb Vasc Biol. 2020;40(2):446–61. 31801372 10.1161/ATVBAHA.119.313226 PMC6975521

[13] Jones PA. Functions of DNA methylation: islands, start sites, gene bodies and beyond. Nat Rev Genet. 2012;13(7):484–92. 22641018 10.1038/nrg3230

[14] Portilla-Fernandez E, Hwang SJ, Wilson R, Maddock J, Hill WD, Teumer A, Meta-analysis of epigenome-wide association studies of carotid intima-media thickness. Eur J Epidemiol. 2021;36(11):1143–55. 34091768 10.1007/s10654-021-00759-z PMC8629903

[15] Dueker ND, Zhao H, Gardener H, Kaur SS, Dong C, Cabral D, Hypermethylation of PM20D1 Is Associated With Carotid Bifurcation Intima-Media Thickness in Dominican Republic Families. J Am Heart Assoc. 2025;14(2):e034033. 10.1161/JAHA.123.034033 PMC12054445 39791430

[16] Gijsberts CM, Groenewegen KA, Hoefer IE, Eijkemans MJ, Asselbergs FW, Anderson TJ, Race/Ethnic Differences in the Associations of the Framingham Risk Factors with Carotid IMT and Cardiovascular Events. PLoS One. 2015;10(7):e0132321. 10.1371/journal.pone.0132321 PMC4489855 26134404

[17] Strategy Group PMsO. Population in Brief 2024. 2024.

[18] Wang X, Mina T, Sadhu N, Jain PR, Ng HK, Low DY, The Health for Life in Singapore (HELIOS) Study: delivering precision medicine research for Asian populations. Nat Commun. 2025;17(1):1. 41469377 10.1038/s41467-025-65774-0 PMC12764833

[19] Sadhu N, Dalan R, Jain PR, Lee CJM, Pakkiri LS, Tay KY, Metabolome-wide association identifies ferredoxin-1 (FDX1) as a determinant of cholesterol metabolism and cardiovascular risk in Asian populations. Nat Cardiovasc Res. 2025;4(5):567–83. 40360795 10.1038/s44161-025-00638-w

[20] Chan SH, Bylstra Y, Teo JX, Kuan JL, Bertin N, Gonzalez-Porta M, Analysis of clinically relevant variants from ancestrally diverse Asian genomes. Nat Commun. 2022;13(1):6694. 36335097 10.1038/s41467-022-34116-9 PMC9637116

[21] Wu D, Dou J, Chai X, Bellis C, Wilm A, Shih CC, Large-Scale Whole-Genome Sequencing of Three Diverse Asian Populations in Singapore. Cell. 2019;179(3):736–49 e15. 31626772 10.1016/j.cell.2019.09.019

[22] Zhang Q, Marioni RE, Robinson MR, Higham J, Sproul D, Wray NR, Genotype effects contribute to variation in longitudinal methylome patterns in older people. Genome Med. 2018;10(1):75. 30348214 10.1186/s13073-018-0585-7 PMC6198530

[23] Lehne B, Drong AW, Loh M, Zhang W, Scott WR, Tan ST, A coherent approach for analysis of the Illumina HumanMethylation450 BeadChip improves data quality and performance in epigenome-wide association studies. Genome Biol. 2015;16(1):37. 25853392 10.1186/s13059-015-0600-x PMC4365767

[24] Houseman EA, Accomando WP, Koestler DC, Christensen BC, Marsit CJ, Nelson HH, DNA methylation arrays as surrogate measures of cell mixture distribution. BMC Bioinformatics. 2012;13:86. 22568884 10.1186/1471-2105-13-86 PMC3532182

[25] Stegle O, Parts L, Piipari M, Winn J, Durbin R. Using probabilistic estimation of expression residuals (PEER) to obtain increased power and interpretability of gene expression analyses. Nat Protoc. 2012;7(3):500–7. 22343431 10.1038/nprot.2011.457 PMC3398141

[26] Zhu Z, Zhang F, Hu H, Bakshi A, Robinson MR, Powell JE, Integration of summary data from GWAS and eQTL studies predicts complex trait gene targets. Nat Genet. 2016;48(5):481–7. 27019110 10.1038/ng.3538

[27] Giambartolomei C, Vukcevic D, Schadt EE, Franke L, Hingorani AD, Wallace C, Bayesian test for colocalisation between pairs of genetic association studies using summary statistics. PLoS Genet. 2014;10(5):e1004383. 10.1371/journal.pgen.1004383 PMC4022491 24830394

[28] Dalan R. Carotid atherosclerosis : an ultrasonographic window for subclinical atherosclerotic cardiovascular disease. Nanyang Technological University: Nanyang Technological University; 2024.

[29] Roadmap Epigenomics C, Kundaje A, Meuleman W, Ernst J, Bilenky M, Yen A, Integrative analysis of 111 reference human epigenomes. Nature. 2015;518(7539):317–30. 25693563 10.1038/nature14248 PMC4530010

[30] Hammal F, de Langen P, Bergon A, Lopez F, Ballester B. ReMap 2022: a database of Human, Mouse, Drosophila and Arabidopsis regulatory regions from an integrative analysis of DNA-binding sequencing experiments. Nucleic Acids Res. 2022;50(D1):D316–D25. 34751401 10.1093/nar/gkab996 PMC8728178

[31] Pidsley R, Zotenko E, Peters TJ, Lawrence MG, Risbridger GP, Molloy P, Critical evaluation of the Illumina MethylationEPIC BeadChip microarray for whole-genome DNA methylation profiling. Genome Biol. 2016;17(1):208. 27717381 10.1186/s13059-016-1066-1 PMC5055731

[32] McAllan L, Baranasic D, Villicana S, Brown S, Zhang W, Lehne B, Integrative genomic analyses in adipocytes implicate DNA methylation in human obesity and diabetes. Nat Commun. 2023;14(1):2784. 37188674 10.1038/s41467-023-38439-z PMC10185556

[33] Tanos BE, Yang HJ, Soni R, Wang WJ, Macaluso FP, Asara JM, Centriole distal appendages promote membrane docking, leading to cilia initiation. Genes Dev. 2013;27(2):163–8. 23348840 10.1101/gad.207043.112 PMC3566309

[34] Engelke MF, Waas B, Kearns SE, Suber A, Boss A, Allen BL, Acute Inhibition of Heterotrimeric Kinesin-2 Function Reveals Mechanisms of Intraflagellar Transport in Mammalian Cilia. Curr Biol. 2019;29(7):1137–48 e4. 30905605 10.1016/j.cub.2019.02.043 PMC6445692

[35] Du F, Peng L, Wang Q, Dong K, Pei W, Zhuo H, CCDC12 promotes tumor development and invasion through the Snail pathway in colon adenocarcinoma. Cell Death Dis. 2022;13(2):187. 35217636 10.1038/s41419-022-04617-y PMC8881494

[36] Bosco B, Rossi A, Rizzotto D, Hamadou MH, Bisio A, Giorgetta S, DHX30 Coordinates Cytoplasmic Translation and Mitochondrial Function Contributing to Cancer Cell Survival. Cancers (Basel). 2021;13(17). 10.3390/cancers13174412 PMC8430983 34503222

[37] Mannucci I, Dang NDP, Huber H, Murry JB, Abramson J, Althoff T, Genotype-phenotype correlations and novel molecular insights into the DHX30-associated neurodevelopmental disorders. Genome Med. 2021;13(1):90. 34020708 10.1186/s13073-021-00900-3 PMC8140440

[38] Siriwardana NS, Meyer R, Havasi A, Dominguez I, Panchenko MV. Cell cycle-dependent chromatin shuttling of HBO1-JADE1 histone acetyl transferase (HAT) complex. Cell Cycle. 2014;13(12):1885–901. 24739512 10.4161/cc.28759 PMC4111752

[39] Zhou MI, Foy RL, Chitalia VC, Zhao J, Panchenko MV, Wang H, Jade-1, a candidate renal tumor suppressor that promotes apoptosis. Proc Natl Acad Sci U S A. 2005;102(31):11035–40. 16046545 10.1073/pnas.0500757102 PMC1182408

[40] Tamai I, Ohashi R, Nezu J, Yabuuchi H, Oku A, Shimane M, Molecular and functional identification of sodium ion-dependent, high affinity human carnitine transporter OCTN2. J Biol Chem. 1998;273(32):20378–82. 9685390 10.1074/jbc.273.32.20378

[41] Ingoglia F, Visigalli R, Rotoli BM, Barilli A, Riccardi B, Puccini P, Human macrophage differentiation induces OCTN2-mediated L-carnitine transport through stimulation of mTOR-STAT3 axis. J Leukoc Biol. 2017;101(3):665–74. 27733576 10.1189/jlb.1A0616-254R

[42] Lu YW, Martino N, Gerlach BD, Lamar JM, Vincent PA, Adam AP, MEF2 (Myocyte Enhancer Factor 2) Is Essential for Endothelial Homeostasis and the Atheroprotective Gene Expression Program. Arterioscler Thromb Vasc Biol. 2021;41(3):1105–23. 33406884 10.1161/ATVBAHA.120.314978 PMC7938420

[43] Xu Z, Yoshida T, Wu L, Maiti D, Cebotaru L, Duh EJ. Transcription factor MEF2C suppresses endothelial cell inflammation via regulation of NF-kappaB and KLF2. J Cell Physiol. 2015;230(6):1310–20. 25474999 10.1002/jcp.24870 PMC4519036

[44] Zhao X, Di Q, Liu H, Quan J, Ling J, Zhao Z, MEF2C promotes M1 macrophage polarization and Th1 responses. Cell Mol Immunol. 2022;19(4):540–53. 35194174 10.1038/s41423-022-00841-w PMC8975968

[45] Gunay-Aygun M, Falik-Zaccai TC, Vilboux T, Zivony-Elboum Y, Gumruk F, Cetin M, NBEAL2 is mutated in gray platelet syndrome and is required for biogenesis of platelet alpha-granules. Nat Genet. 2011;43(8):732–4. 21765412 10.1038/ng.883 PMC3154019

[46] Kahr WH, Lo RW, Li L, Pluthero FG, Christensen H, Ni R, Abnormal megakaryocyte development and platelet function in Nbeal2(−/−) mice. Blood. 2013;122(19):3349–58. 23861251 10.1182/blood-2013-04-499491 PMC3953091

[47] Kahr WH, Hinckley J, Li L, Schwertz H, Christensen H, Rowley JW, Mutations in NBEAL2, encoding a BEACH protein, cause gray platelet syndrome. Nat Genet. 2011;43(8):738–40. 21765413 10.1038/ng.884 PMC6050511

[48] Delage L, Carbone F, Riller Q, Zachayus JL, Kerbellec E, Buzy A, NBEAL2 deficiency in humans leads to low CTLA-4 expression in activated conventional T cells. Nat Commun. 2023;14(1):3728. 37349339 10.1038/s41467-023-39295-7 PMC10287742

[49] Ge J, Jing F, Ji R, Tian A, Su X, Li W, Age-Related Trends in the Predictive Value of Carotid Intima-Media Thickness for Cardiovascular Death: A Prospective Population-Based Cohort Study. Journal of the American Heart Association. 2023;12(13). 10.1161/JAHA.123.029656 PMC10356101 37345827

[50] Deppermann C, Cherpokova D, Nurden P, Schulz JN, Thielmann I, Kraft P, Gray platelet syndrome and defective thrombo-inflammation in Nbeal2-deficient mice. J Clin Invest. 2013;123(8):3331–42. 23863626 10.1172/JCI69210 PMC4011026

[51] Aarts CEM, Downes K, Hoogendijk AJ, Sprenkeler EGG, Gazendam RP, Favier R, Neutrophil specific granule and NETosis defects in gray platelet syndrome. Blood Adv. 2021;5(2):549–64. 33496751 10.1182/bloodadvances.2020002442 PMC7839360

[52] Ishigaki K, Akiyama M, Kanai M, Takahashi A, Kawakami E, Sugishita H, Large-scale genome-wide association study in a Japanese population identifies novel susceptibility loci across different diseases. Nat Genet. 2020;52(7):669–79. 32514122 10.1038/s41588-020-0640-3 PMC7968075

[53] Mishra A, Malik R, Hachiya T, Jurgenson T, Namba S, Posner DC, Stroke genetics informs drug discovery and risk prediction across ancestries. Nature. 2022;611(7934):115–23. 36180795 10.1038/s41586-022-05165-3 PMC9524349

[54] Sakaue S, Kanai M, Tanigawa Y, Karjalainen J, Kurki M, Koshiba S, A cross-population atlas of genetic associations for 220 human phenotypes. Nat Genet. 2021;53(10):1415–24. 34594039 10.1038/s41588-021-00931-x PMC12208603

[55] Consortium GT. The GTEx Consortium atlas of genetic regulatory effects across human tissues. Science. 2020;369(6509):1318–30. 32913098 10.1126/science.aaz1776 PMC7737656

